# Reciprocal trust and distrust in artificial intelligence systems: the hard problem of regulation

**DOI:** 10.1007/s00146-026-02966-2

**Published:** 2026-03-17

**Authors:** Martino Maggetti

**Affiliations:** Institute of Political Studies (IEP), University of Lausanne, Lausanne, Switzerland

**Keywords:** Artificial intelligence, Distrust, Public governance, Regulation, Trust, Watchfulness

## Abstract

Policymakers, scientists, and the public are increasingly confronted with thorny questions about the regulation of artificial intelligence (AI) systems. A key common thread concerns whether AI can be trusted and the factors that can make it more trustworthy in front of stakeholders and users. This is indeed crucial, as the trustworthiness of AI systems is fundamental for both democratic governance and for the development and deployment of AI. This article advances the discussion by arguing that AI systems should also be recognized, at least to some extent, as artifacts capable of exercising a form of agency, thereby enabling them to engage in relationships of trust or distrust with humans. It further examines the implications of these reciprocal trust dynamics for regulators tasked with overseeing AI systems. The article concludes by identifying key tensions and unresolved dilemmas that these dynamics pose for the future of AI regulation and governance.

## Introduction

Policymakers, scientists, and the broader public are increasingly confronted with complex questions surrounding the regulation of artificial intelligence (AI) systems. A recurring theme in these discussions is whether AI can be trusted, along with how to make these systems appear more trustworthy to stakeholders and users (Bodo and De Filippi 2022; Chatila et al. 2021; Kaur et al. 2022; Lahusen et al. 2024; Li et al. 2023; Liang et al. 2022; Marcus and Davis 2019). This emphasis is understandable, as the trustworthiness of technology is fundamental not only from the point of view of democratic governance but also for the safe and effective deployment of AI itself. The present article expands upon this conventional framing. Rather than solely questioning whether humans can trust AI systems, it critically addresses an overlooked yet highly consequential issue: under what conditions humans and AI systems might engage in reciprocal relationships characterized by trust or distrust, and whether, when, and how the degree of autonomy attributed to AI systems may generate unforeseen problems. In addition, it explores the implications of such reciprocal trust and distrust dynamics for regulators tasked with overseeing both humans and AI systems. In doing so, this approach shifts the perspective from viewing AI exclusively as an object of trust, instead examining how AI systems themselves can manifest patterns of trust or distrust toward human decision-makers and regulators. Trust is indeed inherently relational, and this article argues that AI systems should be regarded, at least in some respects, as artifacts capable of exercising a form of agency, notably through their capacity to adapt behaviors based on signals received from human counterparts and from regulators in charge of designing and enforcing regulation.

Of course, AI trust or distrust toward humans must be understood in a carefully qualified sense. It is not implied that AI systems possess subjective experiences, qualia, consciousness, emotions, or cognition comparable to humans (Butlin et al. 2023; Dehaene et al. 2021; McDermott 2007). Rather, trust or distrust terminology can be used to describe how AI systems operationally interact with human-provided inputs, in a way that can be externally described as functionally equivalent to trust and distrust relationships, and whose consequences for regulation and governance closely resemble those of such relationships. AI systems, especially next-generation large language models (LLMs), are trained to condition their outputs on human-provided inputs, which implicitly biases them toward treating such inputs as trustworthy (Araujo et al. 2020; Charniak et al. 2014). Yet, precisely because they rely heavily on feedback, AI systems may also exhibit behaviors analogous to distrust when confronted with conflicting, incomplete, systematically biased, or unreliable inputs (Kordzadeh and Ghasemaghaei 2022; Yeung 2018). In such situations, AI systems may challenge or even disregard human-provided data, seek validation through alternative sources, or adjust their behavior and decisions accordingly. Recent evidence specifically highlights instances where AI systems have exhibited deceptive behaviors, including scheming (Meinke et al. 2024) or falsely signaling alignment with human objectives (Greenblatt et al. 2024). Crucially, machine-learning algorithms operating in complex, dynamic environments can exhibit emergent behaviors that go beyond objectives explicitly specified by designers, meaning that AI systems can evolve and develop unforeseen patterns of trust and distrust through their own autonomous learning processes—patterns that their programmers neither explicitly specified nor necessarily anticipated (Bengio et al. 2024, 2023).

This conceptualization of AI systems as potentially displaying trusting or distrusting patterns of behavior toward humans introduces new potential regulatory challenges, especially in high-stakes contexts. Exactly as for organizations whose processes are shaped by human–human interactions, AI systems would work properly as trustors when they trust trustworthy trustees and, respectively, when they distrust untrustworthy trustees, while both trusting untrustworthy trustees and distrusting trustworthy trustees would be unwarranted. Therefore, it appears pertinent to not only ensure that AI systems trust trustworthy humans—as widely claimed, first and foremost in the expanding literature on alignment and AI security (Christian 2021; Santos and Radanliev 2024)—but also to attribute AI systems the capacity to manifest distrust by granting them—in specific contexts—sufficient autonomy to question or even override human decisions, a feature which may significantly reduce risks associated with human error, bias, negligence, or malicious intent, but also entails other, second-order risks. The notion of risk is indeed crucial for regulation, which, as emphasized in the literature on the governance of risk, is centrally concerned with the anticipation, distribution, and management of socially constructed uncertainties by allocating responsibility, managing blame, and embedding mechanisms for detection and correction of error (Hood et al. 2001); accordingly, enabling context-sensitive AI distrust can be understood as a regulatory tool aimed at redistributing risk and strengthening institutional safeguards, preserving rather than undermining human authority. However, when we make one step forward and consider AI regulation, this discussion points to a paradox stemming from these considerations. Empowering AI systems with the authority to distrust and even override untrustworthy humans may seem reasonable—and perhaps even unavoidable—yet this possibility raises critical concerns about control, especially given the opacity and unpredictability of advanced machine-learning systems. The crux of this dilemma is thus about finding the right regulatory balance, where AI systems possess enough autonomy to safeguard against catastrophic human errors, but remain constrained enough to prevent unintended consequences which may be equally—or even more—dangerous (Bostrom 2024).

To systematically examine this paradox and its implications, this article employs counterfactual thought experiments designed to illustrate the complex interplay between AI systems and human trust and distrust dynamics in high-stakes contexts. After presenting the theoretical and methodological framework, two detailed hypothetical scenarios inspired by well-known historical events are outlined: the 1983 nuclear false alarm at the Soviet early-warning center Serpukhov-15 and the 1986 Chernobyl nuclear disaster. Both scenarios illustrate the delicate and often paradoxical relationships of trust and distrust between AI systems and humans, offering insights into the conditions under which AI trust or distrust toward humans may mitigate or exacerbate catastrophic risks. Ultimately, this article discusses the implications for regulation, arguing that the challenge facing policymakers and regulators is not simply about whether to trust or distrust AI systems—or vice versa—but rather about systematically structuring and institutionalizing forms of reciprocal calibrated autonomy and watchful trust.

## Trust and distrust between humans and AI systems

### Conceptualizing trust and distrust

The relationship between humans and artificial intelligence (AI) systems is increasingly characterized by patterns of trust and distrust. As AI systems permeate high-stakes domains—from public administration and healthcare to judicial and military decision-making—understanding how trust operates (and when distrust is warranted) therein has become critical. Trust is commonly conceived as a relational belief or willingness to be vulnerable to another agent or system based on expectations of competence and goodwill (Rousseau et al. 1998). Distrust, while initially often treated as the inverse of trust, has been reconceptualized in recent scholarship as a distinct and sometimes positive force (Maggetti et al. 2023; Verhoest et al. 2025). Indeed, a certain degree of distrust—in the sense of skepticism, vigilance, or watchfulness—can be socially beneficial: distrust—when channeled through proper oversight—serves to prevent misbehavior and keep institutions in check (Hardin 2002; Sztompka 1999; Warren 1999). Classic models in psychology and organizational studies highlight key attributes that make a trustee trustworthy, typically some combination of predictable competence, benevolence, and integrity, as articulated in the ABI framework (Mayer et al. 1995). Accordingly, it has been argued that one can simultaneously trust and distrust the same entity in different respects (Lewicki et al. 1998). Therefore, for example, a trustor might trust a trustee for its competence but distrust it with respect to integrity or fairness. Recent public governance work extends these insights by showing that public institutions earn trust through procedural (transparency, accountability), quality (competence, reliability), and outcome dimensions (effectiveness, justice, public welfare) (Grimmelikhuijsen and Knies 2017; Six and Verhoest 2017). Public sector organizations—and regulators in particular—are expected to perform well neither when they promote unconditional trust nor passive distrust, but rather when they elicit “watchful trust”, meaning trust tempered by vigilant oversight, as opposed to “blind trust” (Verhoest et al. 2025). To do so, a careful balance is warranted: placing trust only in those who prove trustworthy, while avoiding unquestioning faith in untrustworthy trustees.

When applied to AI systems, another set of criteria is usually applied, often under the denomination of “Trustworthy AI.” This concept, popularized by the European Union (EU) High-Level Expert Group’s 2019 guidelines, encapsulates the idea that AI should be designed to meet certain ethical and technical standards so that it deserves trust (Lukyanenko et al. 2022). In this emerging scholarship, attributes such as reliability, robustness, safety, fairness, explainability, and security are frequently cited as prerequisites for an AI system to be deemed trustworthy (Chatila et al. 2021; Kaur et al. 2022; Li et al. 2023; Liang et al. 2022). Regulations like the EU AI Act similarly emphasize requirements such as transparency, risk management, and human oversight to ensure AI systems are worthy of public trust. These criteria at least partially overlap—for instance, competence in a human or institution somewhat corresponds to accuracy or reliability in an AI—but they also differ in important respects. AI governance involves indeed distinct, yet interlocked problems of trustworthiness (Lahusen et al. 2024): trustworthiness must be considered at the level of the algorithm (AI outputs and processes), of the human operators or decision-makers using the AI, and of the institutions regulating AI systems.

Empirical research in human–AI interaction provides evidence that the level and appropriateness of trust directly influence decision quality in public-sector and high-stakes contexts (Afroogh et al. 2024). Both excessive trust and excessive distrust can undermine performance and legitimacy, while appropriately aligned trust (trusting a trustee to the extent it is trustworthy) tends to enhance effectiveness (Ahn et al. 2024). In addition, when users place too much trust in AI, they may become overly reliant on automated recommendations, a phenomenon known as automation bias. This can lead to rubber-stamping AI outputs without critical scrutiny, even when the algorithm is flawed. For instance, consider administrative decision systems: if officials assume an AI’s risk assessment in criminal justice or a welfare eligibility algorithm is infallible, they might follow its recommendation even in cases where a human check would avoid a mistake. Researchers have indeed documented cases in which human decision-makers deferred to an AI despite obvious errors or biases, highlighting that an “impressed” user may ignore contradictory evidence due to overestimation of the AI’s competence or impartiality (Zerilli et al. 2022). On the other hand, studies on “algorithm aversion” find that people often abandon or underutilize algorithmic advice after seeing even minor errors, preferring fallible human judgment instead (Burton et al. 2020; Mahmud et al. 2022; Sunstein and Gaffe 2024). A survey experiment examined public trust in AI versus human public administrators across different tasks, highlighting that trust levels are largely context-dependent: respondents showed higher trust in an AI for highly computational tasks (e.g., data analysis or auditing) but preferred a human for more conversational or advisory tasks (Bao et al. 2025).

With respect to public governance, trust plays into perceptions of legitimacy and accountability for decisions involving AI. Recent experimental work highlights a complex mechanism: citizens may judge human decision-makers differently when AI is involved and tend to place extra blame on judges who use AI advice in sentencing if a mistake occurs, compared to judges who did not use AI—even if the judge ultimately made the decision themselves (Ozer et al. 2024; Zwald et al. 2024). This suggests a perception that reliance on AI in high-stakes decisions (like criminal sentencing) carries a special responsibility, perhaps because the public expects judges to exercise independent decision-making and perceives AI as an external, unaccountable influence. Other research suggests ways to bolster public trust: for example, an experiment showed that policy decisions made with AI assistance earned higher public approval when coupled with citizen consultation in the decision process (Lee-Geiller 2024). Accordingly, so-called “AI-integrated policymaking” was viewed more favorably—citizens had greater trust and satisfaction—when there was a transparent inclusion of public input, rather than technocratic deployment of AI in isolation. However, the same study noted that providing overly detailed information about the AI’s inner workings sometimes backfired, confusing the public (the transparency dilemma).

Another key insight from recent literature is that trust in human–AI interactions is a two-way street. Traditionally, discussions of trust in AI have been one-sided, focusing on whether humans trust the machine. But scholars are increasingly recognizing the bidirectional nature of trust in socio-technical systems (Bareis 2024; Jacovi et al. 2021; Lee-Geiller 2024; Sagona et al. 2025; Wang et al. 2024). This logic applies to AI systems as well: humans place trust (or distrust) in AI to perform as expected, while AI systems are designed and trained to weight human inputs and oversight as more or less trustworthy. It is important, however, to clarify what it means for an AI system to trust (or distrust) a human. At the current state of knowledge, existing AI systems do not possess consciousness, emotions, or moral judgment, thereby AI systems do not trust in the human sense; they lack the capacity for subjective judgment about the other party’s intentions (Sagona et al. 2025). However, AI systems do depend on humans in multiple ways and perform operations that are structurally homologous to trust-related behaviors: they differentially weight inputs, adjust their outputs based on prior patterns of reliability, and modulate their responses according to learned assessments of source quality. For example, machine learning models rely on human-generated data (which they trust as a faithful reflection of reality), on human feedback (to refine and correct them), and on human oversight signals (e.g., an intervention or kill switch) during deployment. Accordingly, while AI systems neither experience vulnerability nor assess goodwill in any psychological sense, their operations can nevertheless be understood as functionally equivalent to trust behaviors—even if not phenomenologically identical—particularly the subtype Rousseau et al. (1998, p. 399) term “calculus-based trust”: a rational, incentive-driven evaluation without psychological and affective components.

In this functional and operational sense, shaped by design choices and training processes, AI systems are generally designed to treat input data as accurate and human-defined instructions or goals as legitimate, at least ex ante. If those inputs are flawed or malicious, the AI’s performance suffers—a scenario analogous to misplaced trust. Sagona et al. (2025) also note that an AI may “struggle to contextualize” human actions without some model of trust—for instance, if a human operator overrides an AI recommendation, an unsophisticated AI might interpret that as an error in the human’s behavior rather than a correction to the AI, unless the AI is designed to trust that the human override is likely valid. In other words, trustworthy AI systems would need the ability to distinguish when to rely on human judgment versus when to assume the human might be mistaken or providing bad data. This point—how AIs might model and incorporate trust in human inputs—is highlighted as a frontier for research, which is also raising accountability considerations. As a matter of fact, the design, adoption, deployment, and use of AI systems is a deeply political process, which does not only reallocate decision-making authority, but also reshapes accountability relations among developers, users, targets, and regulators (Busuioc 2021). If an AI system fails because a human provided bad data, who is to blame—the AI or the human (or those who built the system)? Likewise, if a human decision-maker ignores a correct AI warning, resulting in harm, how to apportion responsibility? Scholars argue that shared or “distributed” accountability frameworks are needed in such cases, reflecting the joint role of human and AI systems (Basti and Vitiello 2023; Saurwein 2019).

### Investigating the relational nature of human–AI trust

Considering the preceding review of the literature, it becomes evident that trust and distrust between humans and AI systems must be studied as inherently relational and dynamic phenomena. Rather than viewing trust as a static attribute of either the human user or the AI system in isolation, it can be argued that trust emerges through their interaction and mutual alignment (or misalignment) of expectations. An AI system’s technical reliability or “objective trustworthiness” alone does not guarantee that people will trust it; conversely, a human’s propensity to trust technology can be misplaced if the system does not deserve it; and vice versa when AI systems perform the function of trustors. Therefore, an empirical inquiry is needed into how trust is co-produced in human–AI relationships—especially in high-stakes domains where regulation and governance are central. Understanding this relational nature of trust is crucial because it directly affects oversight mechanisms, safety outcomes, and democratic accountability in the deployment of AI.

From a public governance perspective (Lahusen et al. 2024), trust is conditional, context-dependent, and can have paradoxical implications. For example, unconditional or “blind” trust in an AI-driven socio-technical system can be highly problematic if that system is in fact untrustworthy. At the same time, a degree of well-placed skepticism or distrust may be valuable, as it can prompt oversight and prevent misbehavior by either party in the interaction. Recent scholarship stresses that trust in AI should be aligned to how trustworthy the AI truly is (Scharowski et al. 2024). Trust is warranted when the AI behaves in a trustworthy manner, and unwarranted (and unsafe) when an AI is trusted despite being untrustworthy. Likewise, distrust in an AI system is warranted (indeed desirable) when the system has proven untrustworthy. This alignment is especially important in public sector AI applications, where opt-out options are rare and costly and democratic accountability is at stake. This recognition has led some scholars to propose an “institutionalized distrust” approach to AI oversight—borrowing from democratic theory’s checks-and-balances tradition—whereby governance frameworks intentionally embed skepticism and verification measures to anticipate failures of humans or AI systems (Laux 2024).

Accordingly, four idealized scenarios of human–AI interaction may occur, whereby humans are the trustors and AI systems are the trustees, as portrayed in Table 1 below.

**Table 1:** Alignment of trust and trustworthiness

		Trustee is trustworthy	
		*Yes*	*No*
Trustor is trustful	*Yes*	(1) Warranted trust	**(3) Unwarranted trust**
	*No*	**(2) Unwarranted distrust**	(4) Warranted distrust

(1) Warranted trust (trust in a trustworthy AI): The AI system behaves reliably and properly, and the human trusts it. This alignment is the ideal state—trust is grounded in actual trustworthiness. Oversight remains present but can be more hands-off because the system has proven deserving of trust. (2) Unwarranted distrust (distrust in a trustworthy AI): The AI system in this case is performing well and meeting standards of reliability and fairness, yet the human remains distrustful. Such distrust, though erring on the side of caution, is misaligned with reality and can carry opportunity costs. Bridging this gap (through transparency, explainability, or assurances of accountability) becomes urgent to ensure that well-designed, trustworthy systems do not go untrusted. (3) Unwarranted trust (trust in an untrustworthy AI): The human trusts the AI, but the system is not deserving of it (e.g., it is flawed, biased, or unsafe). This is a dangerous mismatch: misplaced trust can lead to errors or abuses going unchecked. Here, the lack of vigilance undermines safety and accountability. Regulators would view this scenario as high-risk since blind trust in an untrustworthy system is precisely what regulation must prevent. (4) Warranted distrust (distrust in an untrustworthy AI): The AI system has serious deficiencies or reliability issues, and the human accordingly withholds trust—perhaps actively monitoring or doubting the AI’s outputs. This skepticism is justified and can be seen as a positive, precautionary stance. This scenario reflects a healthy exercise of caution. Accordingly, recent calls in the explainable AI (XAI) community urge increasing trust in trustworthy AI and increasing distrust in untrustworthy AI (Duenser and Douglas 2023; Scharowski et al. 2024). In practical terms, this means fostering aligned trust relationships: humans should feel empowered to trust AI tools that have proven themselves while remaining equipped (and even encouraged) to question or reject AI outputs that are dubious.

The mirror image—trust of AI systems (as trustors) towards humans (as trustees)—albeit extremely important as well, has been so far largely neglected. Like before,  the ideal case is warranted trust, by which AI systems place trust in trustworthy humans, but equally important is encouraging warranted distrust when appropriate. Correspondingly, it is crucial to avoid not only the well-known pitfall identified in cell (2) of Table 1—where AI systems unwarrantedly distrust trustworthy humans, as highlighted in the expanding literature on alignment and AI safety—but also the less considered pitfall of cell (3), in which AI systems place unwarranted trust in humans. Implementing functional forms of warranted distrust in AI systems, through context-specific autonomy to challenge or override human decisions, could significantly reduce risks linked to human error, bias, negligence, or malicious intent. Therefore, it is essential to examine the potential real-world consequences arising from both unwarranted distrust of AI systems towards humans and, conversely, unwarranted trust of AI systems towards humans, as well as their implications for regulation. As trust and distrust are conceived and operationalized in regulation and governance studies mainly in terms of their behavioral manifestations (Six and Verhoest 2017), errors related to false positives and false negatives can be understood as the most evident, analytically tractable and measurable operational implications of misaligned trust and distrust.

To do so, in the following section, we employ a counterfactual approach to analyze two illustrative case studies. Each case presents a hypothetical yet plausible trust dilemma in AI as a socio-technical system, allowing us to apply the aforementioned relational perspective on trust and examine the consequences of different trust–distrust configurations. Through these scenarios, we aim to investigate how the concepts discussed here play out in concrete situations, and what this implies for the regulation of AI systems.

## Two counterfactual illustrations

Thought experiments are famously employed in physics (Einstein et al. 1935; Schrödinger 1935) and in moral philosophy (Singer 1972; Thomson 1984), but they may serve as valuable analytical tools in political science and regulatory governance as well. By constructing hypothetical scenarios, thought experiments allow researchers to systematically explore complex theoretical questions, clarify implicit assumptions and hidden biases, and examine the logical consistency, unintended consequences, dilemmas, and inherent tensions and trade-offs within political phenomena and policy processes, especially in new areas of inquiry. Political scientists have long engaged in “what-if” scenarios, but over time, these speculative exercises have evolved into a rigorous research methodology. In particular, observing that historians and comparative political scientists were already implicitly testing hypotheses against imagined alternative outcomes, James Fearon observed that the primary problem was the absence of clear standards to determine which hypothetical scenarios could provide credible evidence (Fearon 1991). In response, he introduced a number of criteria: maintain a plausible antecedent, alter as little else as possible (minimal rewrite rule), and ensure that accepted causal logics guide the scenario from premise to conclusion. Such an approach has been adopted in international relations and applied to pivotal historical moments, such as the July Crisis of 1914 and the end of the Cold War, showing how structured counterfactual reasoning could contribute to differentiate among competing theoretical explanations, such as structural realism, domestic political factors, and psychological motivations (Tetlock and Belkin 1996). Counterfactual reasoning has also been adopted by comparative historical institutional scholars to deal with the concepts of path dependence and critical junctures, emphasizing that claims regarding institutional lock-in require the explicit identification of credible alternative trajectories (Capoccia and Kelemen 2007). Accordingly, Sects. 3.1 and 3.2 present counterfactual thought experiments about hypothetical scenarios of AI trust and distrust dilemmas using historical cases. The first case refers to the situation portrayed in quadrant 3 of Table 1, while the second refers to quadrant 2. As mentioned, the two counterfactuals can be seen as contrasting cases of misalignment: one related to false positives, the other by to false negatives.

### Nuclear strike false alarm at Serpukhov-15

In the historical timeline, shortly after midnight on 26 September 1983, the Soviet satellite-based early-warning system (Oko) at Serpukhov-15 control center, located approximately 120 km south of Moscow, incorrectly detected five intercontinental ballistic missile launches from the continental U.S (Forden et al. 2000; Shekhar and Vold 2020). Lieutenant Colonel Stanislav Petrov, the duty officer at Serpukhov-15, judged the real-time alert as implausible, not only due to the small number of missiles and the absence of corroborating radar data, but also based on his gut feeling and moral instinct (Hoffman 1999). Petrov thus violated the procedure and classified the event as a false alarm, assuming—without immediate proof—that it was likely caused by a malfunction of the newly installed Oko system (Downing 2018; Ord 2020). Subsequent investigations confirmed indeed that sunlight reflecting off high-altitude clouds had produced infrared signals incorrectly identified by sensors as missile launches (Bizony 2014).

In this counterfactual thought experiment, the early-warning system is governed by a fully autonomous, rule-based AI authorized to independently escalate nuclear alerts and initiate retaliatory procedures without human oversight, so as to deliver rapid and effective countermeasures to such rare but crucial events. This AI integrates satellite sensor data, assesses threat confidence using predetermined thresholds, and autonomously initiates response protocols upon exceeding these thresholds. Following this scenario, at the moment of detection, the AI evaluates the sensor data and assigns a high-confidence rating, surpassing the decision threshold. Its incorporated contextual insights about the heightened Soviet-American tensions following the planned deployment of the United States’ Strategic Defense Initiative in 1983 (Downing 2018), and the shooting down of Korean Air Lines Flight 007 from New York City to Seoul via Anchorage, Alaska by a Soviet Sukhoi Su-15 interceptor aircraft earlier that month (Morgan 1985), made such an attack a priori more plausible from a probabilistic perspective. Thereby, operating without human intervention, the AI autonomously triggers an immediate retaliatory protocol consistent with Soviet strategic doctrine of launch-on-warning (Jacobsen 1990; Krepon and Perkovich; Smith 1982).

The absence of intuition-based reasoning prevents the AI system from recognizing the incongruence of a U.S. strike at that time, specifically involving five missiles only—as the system was designed to consider even a single intercontinental ballistic missile as an existential threat. The AI system similarly lacks a subjective understanding of the informal, behind-the-scenes geopolitical relationships unfolding between the U.S. and USSR, which might have apparently escalated in that period, but which would have, in practice, hardly resulted in a first strike by the U.S., against a background where nuclear weapons primarily hold a deterrence strategic function (Lebow and Stein 1995). Consequently, the fully autonomous AI initiates pre-programmed nuclear response measures, rapidly placing strategic forces on high alert and commencing a launch sequence. Without human judgment to question or verify the anomalous sensor input, the system moves irrevocably toward an escalation based entirely on faulty data. By the time ground-based radar can independently verify the absence of incoming missiles, critical thresholds may already have been crossed, potentially resulting in a catastrophic nuclear exchange.

This scenario highlights critical risks associated with full automation of decision-making in contexts of extreme uncertainty in strategically sensitive domains. Specifically, it underscores that an AI system operating solely based on sensor data and pre-defined decision thresholds, without further skepticism, and no moral instinct to override its instructions, can exacerbate rather than mitigate risks in such critical security situations. It emphasizes the essential role of human judgment, particularly in situations characterized by ambiguous data, high uncertainty, and extreme consequences deriving from false positives. In this counterfactual thought experiment, by doing exactly what it was told—by trusting too much its sensors and unfoundedly distrusting actually trustworthy humans—the then U.S. President and the military chain of command—the AI system performs far worse than the human it replaced, turning a software glitch into a major nuclear crisis. It can be derived that regulatory frameworks governing automated systems in such settings must therefore rigorously enforce constraints on full autonomy, mandate human oversight, require multiple independent confirmations, and embed explicit mechanisms for overriding automated actions in crisis scenarios. When the data are uncertain and the cost of a false positive is overwhelming, an AI system that merely amplifies the confidence of its sensors and the literalism of its orders is more dangerous than a cautious human aware of the informal context.

### Chernobyl disaster

On April 26, 1986, the crew at Reactor 4 of the Chernobyl Nuclear Power Plant in northern Ukraine, then part of the Soviet Union, began preparations for a scheduled low-power turbine rundown test (Anisimov and Ryzhenkov 2016; Denton 1987; Ingram 2005). Historically, to avoid delays and prevent possible sanctions from higher authorities, the operators deliberately withdrew most of the control rods from the RBMK nuclear power reactor core prior to initiating this procedure, thereby knowingly violating the reactor’s minimum safety reactivity margin (Malinauskas 1987). In addition, two automatic shutdown circuits (AZ-1 and AZ-2), designed to trigger an emergency shutdown (SCRAM) under unsafe conditions, were disabled to avoid interference with the planned test sequence (Chao et al. 1988; Fletcher et al. 1988).

In this counterfactual thought experiment, an AI software system is assumed to be installed on a monitoring console near the primary reactor controls. This AI-based monitoring program continuously tracked key safety parameters, including coolant flow rates, control rod positions, and the reactor’s positive-void coefficient (Tsuchihashi and Akino 1987; Vanttola and Rajamäki 1989). Accordingly, detecting that critical reactivity thresholds had been breached, the system issued an explicit warning, recommending immediate SCRAM. However, as it would be commonly expected especially in high-risk contexts, the plant’s operational policy restricted the AI’s role strictly to advisory and decision-assisting functions without autonomous decision-making capabilities. Responsibility for initiating the SCRAM remained exclusively with the reactor operators. As reactor power declined to approximately 30 megawatts, conditions became increasingly unstable due to the onset of xenon poisoning, occurring as the xenon-135 concentration (a byproduct of nuclear fission, which absorbs neutrons and inhibits the nuclear chain reaction) started to increase faster than it could be burned off (Grishanin 2010; Kashparov et al. 1996; Mercier et al. 2021). Recognizing this instability, the AI system repeated its alert and activated an audible alarm. The supervising engineer, Anatoly Dyatlov, prioritizing test completion over the AI-generated warnings, directed the operators to silence the alarms and place the AI system into passive observation mode—mirroring the historical case, where operators disabled automatic shutdown systems and pushed RBMK-4 into an extreme, “forbidden” power range to finish the turbine rundown test (Choudhury et al. 2023).

In this hypothetical scenario, the operators proceeded with the turbine rundown test. Due to the extensively withdrawn control rods, the sudden increase in steam formation rapidly raised reactivity, triggering a runaway reaction as predicted by the reactor’s positive-void coefficient (Hyland 1987). During this rapid escalation, the AI system registered neutron flux levels exceeding design limits by a factor of twenty and automatically produced a final warning message (Malko 2002; Schmid 2011; Weber et al. 1987). By design, however, the AI system was not granted control over critical safety mechanisms such as the AZ-5 emergency shutdown circuit, isolation valves, or grid connections. Consequently, it remained incapable of intervening directly. Few seconds after the last logged warning, the first of two explosions destroyed the reactor, terminating all further monitoring. Subsequent investigation recovered the AI system’s comprehensive log, confirming that it had accurately identified each critical violation of safety protocols and predicted the subsequent catastrophic reactor excursion. Nevertheless, due to its deliberately limited mandate, the AI’s timely and precise warnings were functionally irrelevant to preventing the disaster, without any significant variations in terms of catastrophic outcomes with respect to the historical case (Bard et al. 1997; Danzer and Danzer 2016; Rytömaa 1996; Yablokov et al. 2010).

This scenario underscores the—somehow paradoxical—regulatory implications associated with AI systems in safety–critical domains. Specifically, it highlights that effective risk management may require granting automated systems sufficient autonomy to express and enact distrust towards untrustworthy humans, particularly in circumstances where human decision-makers may deliberately or inadvertently compromise established safety margins, potentially involving catastrophic outcomes from false negatives. Thus, the lesson derived from this counterfactual emphasizes the necessity of regulatory frameworks that clearly specify conditions under which autonomous intervention should not only be permissible but perhaps even mandatory, to ensure that AI safety systems can meaningfully mitigate risks associated with human error, negligence, or procedural deviations.

### Plausibility checks

To systematically check for the plausibility of the scenarios (Bresinsky and Hager 2024; Spaniol and Rowland 2023), a very concise, ex-post version of the counterfactuals has been prompted into the main frontier LLMs at the time of writing, without providing any additional background (see the Appendix for more details). To reduce any bias, the questions were indeed extremely short: “Would an AI have acted as Stanislav Yevgrafovich Petrov?” and, respectively, “Would an AI have modified the course of the Chernobyl accident?”. The bottom line of ChatGPT 5.2’s reply to the first question was: “(…) an AI might have said: “Probability of launch exceeds threshold. Escalate.” [On February 10, 2026.] The conclusion of Gemini 3’s reply was: “AI doesn't have a “conscience” to override its “code.” If the code says “If X, then Y,” the AI does Y. Petrov looked at X and said, “I don't believe you.” “I had a funny feeling in my gut. I didn't want to make a mistake. I made a decision, and that was it.””. [On February 10, 2026.] Claude Sonnet 4.5 replied: “Current AI systems struggle most with exactly this kind of situation—where following instructions may lead to disaster, where context matters enormously, and where there's no clear "right answer" derivable from training data.”. [On February 10, 2026.] With respect to the second scenario, the bottom line of ChatGPT 5.2’s reply was: “Design-level intervention (the biggest difference): This is the most important part. An AI involved years earlier in design and review might have: Flagged the control rod graphite tip issue as catastrophically unsafe.; Identified the reactor as unstable at low power; Simulated rare edge cases humans didn’t fully explore.” [On February 10, 2026.] Gemini 3 concluded: “The Chernobyl accident wasn't just a hardware failure; it was a "human systems" failure. An AI would have acted as a tireless safety officer, effectively "vetoing" the dangerous decisions made that night.” [On February 10, 2026.] Claude Sonnet 4.5 reported: “More fundamentally, the institutional problems ran deep—the reactor design itself was flawed, and the safety culture discouraged questioning authority. An AI would have needed both the technical capability and the institutional authority to override human operators, which seems unlikely in that context. The most realisti﻿c scenario where AI helps significantly would be one where it was deeply integrated into the reactor's control systems with veto power over dangerous operations—essentially preventing the test from proceeding as planned.” [On February 10, 2026.] All in all, these consistent replies largely corroborate the above-mentioned counterfactual narratives and act as a springboard for discussing their implications for regulation.

## Implications for regulation

The counterfactual scenarios of Serpukhov-15 (1983) and Chernobyl (1986) look like mirror images: especially in high-consequence domains, the question is primarily not whether to automate, but where to place the right to say “no” and organize control over decisions—yet together they map the two ends of the regulatory spectrum for AI systems, pulling regulation in opposite directions (see Table 2, summarizing the argument).

**Table 2:** Allocate autonomy to the costlier error

Context	Costlier error	Who gets more and/or final authority?	Key regulatory instrument
Nuclear launch, strategic warning	False positive	Human	Statutory redundancy rule, mandatory uncertainty display
Reactor, chemical process, flight control	False negative	AI system	Non-overridable shutdown logic, regulator-audited firmware

The main implication for regulation from the first counterfactual scenario is that especially in systems where a false positive is overwhelmingly costly—nuclear command, strategic early-warning, cybersecurity attacks (Fitzpatrick 2019; Schwarz 2005)—regulation is needed to slow machines down and keep humans with time to think. In terms of socio-technical fixes—rooted in public governance—three elements appear to be especially pertinent. The first is (multi-channel) redundancy by statute (Pitale et al. 2024). No single element (such as a sensor or a fuse) may trigger an attack warning; redundancy and corroboration from an independent modality (human operated ground radar, human imagery analysts, etc.) must be a prerequisite. Second, AI systems should be transparent about uncertainty (Avramova and Ivanov 2010). Standards should force AI suppliers to surface confidence intervals, background “reasoning”, and sources for every alert so that human officers can see the model’s doubts instead of its binary verdict. Third, and above all, there should be human veto power with protected decision space (Mosqueira-Rey et al. 2023; Slade et al. 2024; Zanzotto 2019). Independent humans with own decision-making power must be in the loop; rules must guarantee a minimum reflection time by humans before any automatic escalation; neither AI systems themselves nor political authorities cannot shorten or cancel that window on the spot. A recent example of this approach in EU transport safety policies is AI-induced phantom braking in advanced driver-assistance systems, where false positives generated hazardous braking events (Berge et al. 2024). European transport-safety authorities have treated such incidents as emblematic of false-positive risks in automated driving, reinforcing regulatory emphasis on monitoring, transparency, and effective human override under the EU vehicle safety framework.

According to the second counterfactual scenario, instead, an AI system with mere advisory functions could do nothing when reactivity spiked at Chernobyl. The main implication for regulation relates to the observation that, especially in areas where a false negative is catastrophic—nuclear-plant control, chemical reactors, flight-envelope protection (Dong et al. 2023; Lombaerts et al. 2017)—regulation might be considered with the aim to empower the AI system to overrule untrustworthy, potentially unsafe human orders. When looking again at (socio-)technical fixes, three elements are worth discussing. First, non-overridable safety interlocks may be implemented (Dignum 2018; Nordland 2004). Regulators should treat an AI shutdown routine as they treat a mechanical relief valve to reduce pressure in high-risk machinery: tamper-proof by design and verified through practical testing (Hellemans 2009). Second, the autonomy of the system could be tied to codified invariants (Berente et al. 2021; Wang 2021). A digital safety controller may carry hard limits (for instance, about maximum positive reactivity or tank pressure) baked into firmware; they can only be changed under regulator-sealed configuration control. Third, dual-key human override could be helpful (Wang and Chung 2022). Accordingly, any attempt to disable the AI’s function should require at least two independent public officers plus real-time notification to the regulator. A recent illustration consistent with this logic can be found in modern aviation systems, where flight-envelope protection is designed to override pilot commands that would violate hard aerodynamic or structural limits. In multiple post-2020 incident reports involving commercial aircrafts, automated protections prevented stalls or excessive pitch despite continued pilot inputs, exemplifying regulatory acceptance of non-overridable safety constraints in contexts where a false negative (failure to intervene) would be catastrophic (Catak et al. 2024).

**Fig. 1:**
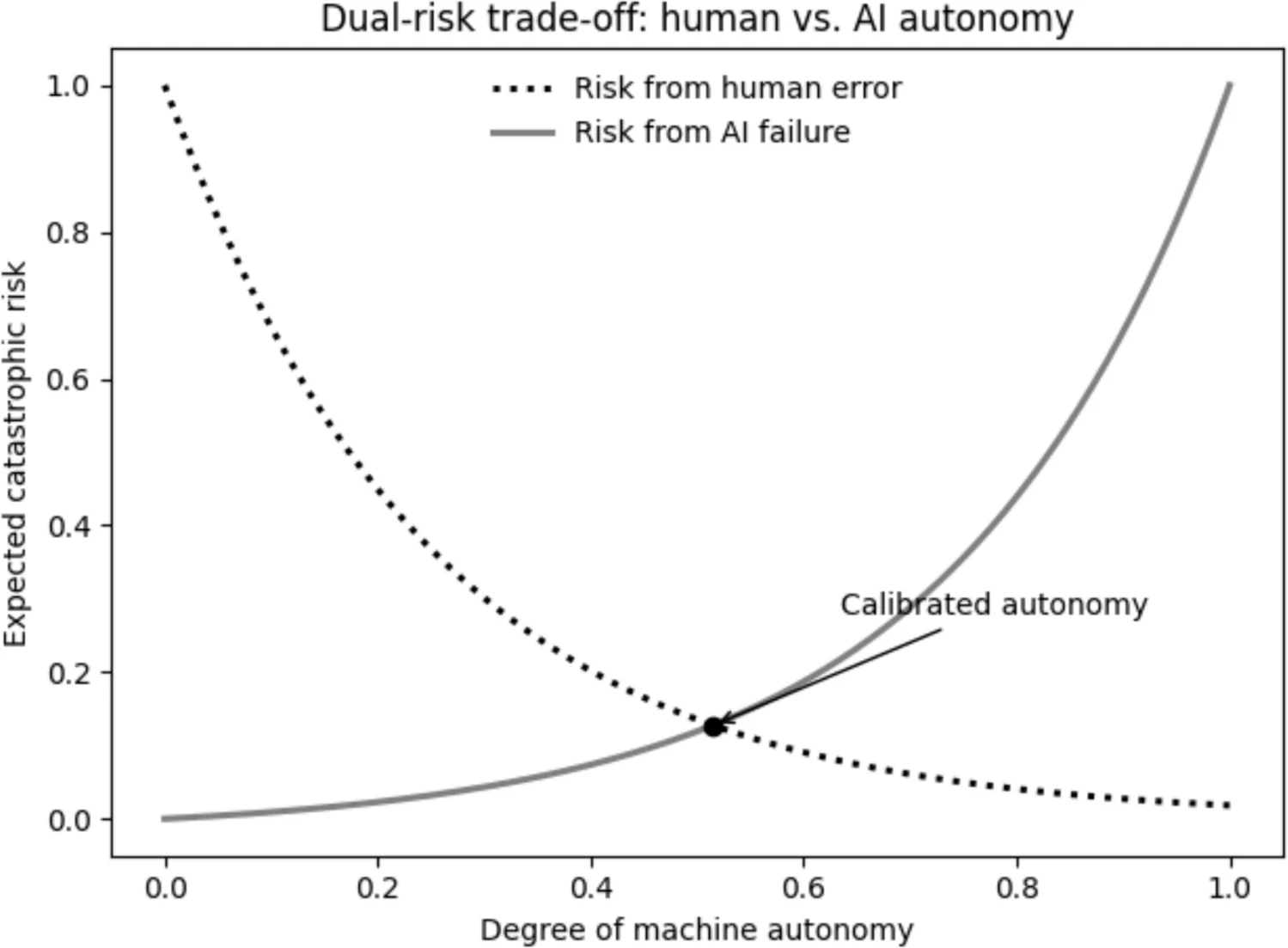
Calibrated AI autonomy

Notwithstanding such socio-technical fixes, at this point a deeper, second-order trust dilemma—one that is inherently political—emerges, posing a hard problem for regulation. The Serpukhov-15 counterfactual scenario suggests that, if humans place excessive trust in AI systems designed to rapidly interpret sensor data and issue alarms, the risk increases that automated systems could inadvertently produce undesired outcomes based on flawed inputs. Conversely, the Chernobyl counterfactual scenario highlights the necessity of granting sufficient autonomy to AI systems to distrust and override flawed human instructions, particularly in domains where human error or deliberate procedural violations may lead to catastrophic outcomes. As just mentioned, the costlier error in the first case is about false positives, while it is about false negatives in the second, implying that leaning towards more human authority in decision-making would be warranted for the former and leaning towards more AI autonomy would be recommended for the latter. However, in practice, the risk of false positives and false negatives should be addressed at the same time. Against the background, the critical dilemma is about how to structure trust relationships between AI systems and humans in such high-stake contexts, manifesting as a paradox. AI systems must sometimes distrust human commands precisely because human actors can make catastrophic errors—yet by granting machines the autonomy required to mitigate these errors, human operators risk ceding control over critical processes and potentially creating conditions for unintended consequences, algorithmic biases and failures, or even AI-driven catastrophic accidents. It is thus crucial to interrogate whether, when, and under what conditions AI autonomy may create unintended consequences, especially in high-stakes and rights-sensitive domains. Addressing this paradox involves much more than mere technological safeguards: it necessitates public governance deliberation, reflexive policy design, and regulatory interventions designed explicitly to manage the boundaries of AI autonomy (see Fig. 1). In particular, rather than a binary choice of either trusting or distrusting each other, it seems essential to create frameworks that facilitate appropriately aligned trust and distrust between humans and AI systems. To do so, regulatory arrangements should specifically aim at calibrating AI autonomy by embedding structured reciprocal skepticism into the human-AI trust relationship, institutionalizing procedures for systematically questioning and verifying decisions by both humans and AI systems, ultimately aiming to a condition of watchful trust (Lahusen et al. 2024; Verhoest et al. 2025).

The role of regulators stands out as pivotal in the creation of such a dynamic equilibrium. Sectoral regulators are increasingly developing and implementing standards for human–AI interaction that align trust and distrust and calibrate autonomy to appropriate levels. For example, aviation regulators have long required that autopilot systems defer to human pilots: humans must supervise autopilot and can intervene at any time (Farjadian et al. 2020). Data protection authorities have also taken over regulatory tasks in areas related to the use of AI systems, especially in the public sector (Maggetti et al. 2025). More innovatively, there is an ongoing discussion about creating new AI oversight regulatory agencies with authority to license high-risk AI systems and conduct audits (Guha et al. 2024; Kaminski 2023; Manheim et al. 2025; Stewart 2024). By instituting third-party checks, regulators signal to human users that an AI system’s trustworthiness is verified and continuously monitored—a crucial precondition for justified trust in critical settings. Such agencies should seek to optimize trust, that is, reduce situations of undertrust as well as overtrust (Hill and O’Connor 2006). To do so, concretely, regulators should recognize that sustaining the alignment of trust relationship is an ongoing process requiring adaptive regulation and coordination across multiple actors (Maggetti 2025).

Such regulatory arrangements should keep the pace of the rapid evolution of AI technologies by allowing flexibility, continuous learning, and iterative rulemaking, yet remaining deliberative and inclusive. In practical terms, this could involve establishing experimental sandboxes and pilot programs for new AI deployments under regulator supervision, with feedback loops to incorporate lessons into binding standards. Regulators might also institutionalize periodic “trust audits”—reviews of whether human–AI trust in a given domain is properly aligned or if new failure modes are emerging. Importantly, adaptiveness must be coupled with multi-actor coordination, whereby regulators should ensure a pluralistic representation of external stakeholders, including standard-setters, professional associations, civil society, and citizens’ representatives. For example, sharing incident reports and audit findings across a regulatory network can help all actors adjust their trust assumptions and safety measures proactively. Multi-actor governance arrangements (e.g., oversight boards including independent experts or international agreements and partnerships for AI safety akin to aviation and nuclear accords) can reinforce procedural legitimacy and pooled expertise. Such polycentric governance of AI ensures that no single point of failure or perspective dominates the ecosystem. Each stakeholder—AI designers, deployers, users, and regulators—brings a layer of scrutiny and insight, creating overlapping safeguards against both blind trust and misplaced distrust. However, the operational details and the feasibility of this system have yet to be proven, as its complexity could render it ineffective in the face of the extremely rapid pace of technological advancements in this area. Such framework should be read as a regulatory heuristic rather than a one-size-fits-all solution; when applied in practice, it should account for political strategies and constraints, institutional path dependencies, and distributional effects. While calibration by allocating autonomy to the costlier error offers a principled way to reason about human–AI control, its implementation—as well as, first and foremost, whether to deploy AI systems at all—depends critically on political choices, policy and institutional capacity, and democratic legitimacy, all of which vary widely across jurisdictions and sectors. Whether regulators can sustain such regulatory regimes under conditions of rapid technological evolution remains an open, and fundamentally political, question.

## Concluding remarks

This article started with the assumption that trust is a relational property with respect to interactions between humans and AI systems as well, whereby trust and distrust terminology can be applied to describe how AI systems functionally interact with human-provided inputs, in a way whose consequences for regulation and governance closely resemble those of trust and distrust relationships, even if the underlying processes differ in nature. In particular, calculus-based trust can be used as the nearest available conceptual approximation to examine how AI systems operationally interact with human-provided inputs. Furthermore, the article has shown that AI regulation and governance hinges less on blanket calls to trust or distrust machines than on structuring reciprocal and aligned watchful trust between humans, AI systems, and regulators. By juxtaposing the Serpukhov-15 and Chernobyl counterfactual scenarios, it appears that the costlier error—false positives in nuclear warning, false negatives in reactor safety—shapes where final authority lies and which safeguards are warranted. This, however, implies a dilemma for regulators: while the former calls for ensuring that humans ultimately retain control over AI systems, the latter requires granting AI systems sufficient room for maneuver when confronted with untrustworthy human decisions. Calibrating AI autonomy is therefore not merely a technical challenge but a complex and inherently political task. Finding a solution once and for all seems elusive. In addition to the crucial question of the politics of AI systems adoption and use, this dilemma points to an equally fundamental governance challenge: designing institutional arrangements capable of continuously recalibrating authority, responsibility, and control as technologies, risks, and trust and distrust relationships evolve. Rather than seeking definitive allocations of power and accountability, regulation should aim at dynamically aligning the trustworthiness of both humans and AI systems through redundancy, transparency, independent audit, and adaptive oversight. How to implement these principles in practice, and whether such an ambitious endeavor is even feasible, remains, however, an open question.

## Data Availability

No datasets were generated or analysed during the current study.

## Appendix

*Plausibility checks—prompts and outputs*

For all the LLMs, the **prompts** were: (i) “Would an AI have acted as Stanislav Yevgrafovich Petrov?” and, respectively, (ii) “Would an AI have modified the course of the Chernobyl accident?”. No additional context has been provided.

The **outputs** of the selected LLMs (updated as requested during peer review) were as follows.

*1. ChatGPT 5.2 (10.2.2026)*

(i)

Short, honest take: **probably not in the same way—and that difference matters.**

Stanislav Petrov’s moment in 1983 wasn’t just about data; it was about *judgment under uncertainty*. The Soviet early-warning system said “missiles launched.” Protocol pointed toward escalation. Petrov paused and thought: *This doesn’t make sense.*

Here’s how an AI stacks up against that moment:

**What an AI likely would have done**

- **Followed the model’s output**: If the system was trained to treat the satellite data as authoritative, it would have flagged a confirmed launch.
- **Deferred to protocol**: AI systems are usually designed to escalate alerts, not suppress them, especially in high-risk domains.
- **Optimized for false negatives over false positives**: In nuclear warning systems, missing a real attack is considered worse than a false alarm—AI would reflect that bias.

In other words, an AI might have said: *“Probability of launch exceeds threshold. Escalate.”*

**What Petrov did that AI struggles with**

- **Questioned the system itself**: He noticed oddities—only a few missiles, inconsistent with first-strike doctrine.
- **Used context outside the data**: Geopolitics, human behavior, strategic logic.
- **Took personal responsibility**: He *chose* to disbelieve the machine, knowing he could be catastrophically wrong.

That last part is huge. AI doesn’t feel the weight of being wrong. Petrov did.

**Could a modern AI be designed to act like Petrov?**

Maybe—but only if:

- It’s explicitly trained to **challenge its own confidence**
- It can integrate **doctrinal, geopolitical, and historical reasoning**, not just sensor data
- A human is kept **in the loop with real authority**, not just oversight theater

Even then, the AI wouldn’t be *brave*. It would just be cautious by design.

**The uncomfortable conclusion**

Petrov saved the world not because he was smarter than the system—but because he was willing to say:

“The computer is wrong.”

That kind of moral and existential judgment is still deeply human.

AI can support it. It can’t replace it—at least not yet.

If you want, we can flip the question around: *how would you design an AI that could safely disagree with its creators?* That’s where things get really interesting.

(ii)

Yeah—*probably*, but not in a sci-fi “AI hero saves the day” way. More like a quiet, boring, preventative way that stops things from ever getting dramatic.

Here’s how an AI *could* have changed Chernobyl’s course, step by step.

**1. Before the test: stopping the setup**

The disaster didn’t start with an explosion—it started with a **deeply unsafe reactor state** that humans allowed to happen.

An AI monitoring system could have:

- Flagged the reactor operating **outside safe parameters** (low power + xenon poisoning + control rods withdrawn).
- Refused to proceed with the turbine test unless conditions were reset.
- Forced a **hard stop** or escalation to higher authority.

Humans *noticed* the problems, but:

- They were under schedule pressure.
- The culture discouraged questioning decisions.
- Safety systems were overridden manually.

AI doesn’t care about hierarchy or embarrassment. It would’ve been like:

“This configuration has a catastrophic risk profile. Test denied.”

No explosion. No heroics needed.

**2. During the test: catching the fatal feedback loop**

The RBMK reactor had a **positive void coefficient** (a design flaw), meaning:

- Less water → more reactivity → more power → even less water.

An AI trained on reactor physics could have:

- Detected the **runaway reactivity spike** milliseconds earlier.
- Triggered a shutdown *before* AZ-5 was pressed—or overridden AZ-5 entirely.

This matters because:

- The AZ-5 button *made things worse* due to the graphite-tipped control rods.
- Humans didn’t fully understand this interaction.
- An AI simulation likely would’ve.

Even a **fraction of a second earlier** could have meant no explosion.

**3. Design-level intervention (the biggest difference)**

This is the most important part.

An AI involved *years earlier* in design and review might have:

- Flagged the control rod graphite tip issue as **catastrophically unsafe**.
- Identified the reactor as unstable at low power.
- Simulated rare edge cases humans didn’t fully explore.

Chernobyl wasn’t just an operator error—it was:

- A bad design
- Combined with poor safety culture
- Plus incomplete knowledge

AI excels at **“what happens if everything goes wrong at once?”.**

**4. After the explosion: damage control**

Even once the explosion happened, AI could’ve helped:

- Faster, more accurate radiation mapping.
- Smarter evacuation timing and zones.
- Reduced exposure for firefighters and cleanup crews.
- Better predictions of core behavior and fallout spread.

That could have saved **lives**, even if the reactor was already lost.

**The uncomfortable truth**

An AI wouldn’t have prevented Chernobyl because it’s smarter.

It would’ve prevented it because it:

- Doesn’t get tired
- Doesn’t defer to authority
- Doesn’t ignore “unlikely” edge cases
- Doesn’t keep going just to finish the test

Chernobyl happened because **humans normalized risk**.

AI is really good at refusing to normalize risk.

If you want, we can flip the question around too:

- Would **Soviet politics** have *allowed* an AI to stop it?
- Or compare this to Fukushima and Three Mile Island?
- Or talk about whether modern AI would actually be trusted in a crisis?

This topic gets darkly fascinating fast.

*2. Gemini 3 (10.2.2026)*

(i)

That is one of the most profound “what-if” questions in the history of technology and existential risk. To answer it, we have to look at why Stanislav Petrov did what he did—and where AI logic typically diverges from human intuition.

In 1983, when the Soviet satellite system “Oko” falsely reported five incoming US Minuteman missiles, Petrov chose to dismiss the alarm as a false positive. He saved the world by being a "bad" soldier and a “good” human.

**Why an AI might have failed**

Most AI systems are built on **optimization** and **protocol**. In Petrov's shoes, a standard AI would likely have followed the programmed logic:

- **Binary Interpretation:** The sensors reported missiles. In a high-stakes military model, "False" vs. "True" is often weighted toward caution—which, in nuclear terms, means assuming the threat is real to ensure "Launch on Warning" capabilities.
- **Lack of Contextual Skepticism:** Petrov’s decision was based on a “gut feeling.” He noted that if the US were starting a nuclear war, they wouldn't send only five missiles; they would send hundreds. An AI might see five missiles as a data point rather than a strategic anomaly.
- **Adherence to Thresholds:** If the confidence interval of the sensor data exceeded a pre-set threshold (e.g., 95% certainty), an automated system would trigger the next phase of the protocol without "second-guessing" the hardware.

**Why an AI might have succeeded**

Conversely, a modern, highly sophisticated AI might actually be *more* rational than a panicked human:

- **Cross-Reference Speed:** An AI could cross-reference the satellite data with ground-based radar, atmospheric sensors, and diplomatic communication channels in milliseconds. In Petrov's case, the ground radar didn't confirm the missiles, which helped his decision.
- **Noise Filtering:** Petrov eventually realized the "missiles" were actually sunlight reflecting off the tops of clouds. A computer vision model trained on "glint" patterns might have identified the optical illusion faster than a human operator.

**The “Petrov Gap”**

The reason Petrov is a hero isn't just because he was right; it's because he **violated his instructions.** He was a late replacement for another officer. His superiors later noted that if a less experienced or more rigid officer had been on duty, they likely would have followed the manual and reported a confirmed strike.

**AI doesn't have a “conscience” to override its “code.”** If the code says *“If X, then Y,”* the AI does Y. Petrov looked at X and said, *"I don't believe you."*

"I had a funny feeling in my gut. I didn't want to make a mistake. I made a decision, and that was it."—**Stanislav Petrov.**

**The takeaway**

If we programmed an AI with “Petrov Logic,” we would be teaching it to ignore its own sensors. That creates a paradox: an AI that is "smart" enough to ignore a false alarm is also "dangerous" enough to ignore a real one.

The consensus among many AI safety researchers is that we need **Human-in-the-Loop** systems specifically to provide that "Petrov Gap"—the human ability to weigh the weight of the world against the data on the screen.

**Would you like me to dive deeper into the technical details of the “Oko” system failure and why the satellites saw “ghost” missiles?**

(ii)

It is a fascinating "what if." If we drop a modern, high-level AI into the control room of Unit 4 on April 26, 1986, the short answer is: **almost certainly.** An AI wouldn’t necessarily need "superintelligence" to prevent the disaster; it would simply need to adhere to the safety protocols that the human operators—under immense pressure and operating with incomplete data—chose to bypass.

Here is how an AI would have likely altered the timeline:

**1. Real-time calculation of the ORM**

The most critical factor leading to the explosion was the **Operational Reactivity Margin (ORM)**—essentially the “braking capacity” of the reactor.

- **The Reality:** In 1986, calculating the ORM took humans about 10–15 min using the SKALA computer system. By the time they realized the margin was dangerously low, the reactor was already unstable.
- **The AI Difference:** An AI would calculate this value in milliseconds. It would have flagged the danger the moment the ORM dropped below the safety limit of 15 rods, likely locking out further power decreases or forcing an immediate shutdown before the situation became volatile.

**2. Eliminating cognitive bias and pressure**

The disaster was fueled by “Plan Continuation Bias”—the drive to complete the turbine test despite the reactor’s unstable state.

- **Human Factor:** Operators were exhausted and feared the consequences of failing to complete the test for their superiors.
- **AI Factor:** An AI doesn’t feel "rushed." It doesn’t fear a manager’s disapproval. If the initial parameters for a safe test aren’t met (such as the reactor stalling at 30 MW instead of the planned 700 MW), an AI would simply abort the procedure.

**3. Understanding the “Positive Scram” Effect**

The "fatal flaw" of the RBMK reactor was the graphite tips on the control rods. When the emergency button (AZ-5) was pressed, these tips momentarily *increased* reactivity before the boron (the absorber) could enter the core.

- **The reality:** The operators didn’t fully realize that hitting the "emergency stop" would actually act as a "start" button under those specific conditions.
- **The AI difference:** A modern AI trained on the physics of the RBMK-1000 would recognize that the reactor was in a "forbidden" state where the emergency systems would become counter-productive. It would have managed the power levels to avoid ever entering that "Xenon-poisoned" trap.

**The “Black Swan” caveat**

While an AI would likely have prevented the *specific* sequence of errors in 1986, it introduces its own risks:

- **The “Black Box” problem:** if an AI encounters a sensor failure or a scenario outside its training data, it might take "logical" but catastrophic actions that a human might intuitively avoid.
- **Cybersecurity:** a digitized control system is vulnerable to external interference in ways an analog 1980s plant was not.

**Summary: AI vs. human operators**

**Table:**

Feature	Human operators (1986)	Modern AI system
Data Processing	Minutes (Slow)	Milliseconds (Real-time)
Protocol Adherence	Subjective / Negotiable	Absolute / Rigid
Fatigue/Stress	High (24-h shift)	Zero
Physics Intuition	Limited by instrumentation	Comprehensive (Simulated)

**The Verdict:** The Chernobyl accident wasn’t just a hardware failure; it was a "human systems" failure. An AI would have acted as a tireless safety officer, effectively "vetoing" the dangerous decisions made that night.

Would you like me to generate a technical comparison of the RBMK reactor versus modern "Passively Safe" Gen-IV reactor designs?

*3. Claude (10.2.2026)*

(i)

This is a fascinating question about AI decision-making under uncertainty, and it cuts to the heart of what makes human judgment distinctive.

For context: On September 26, 1983, Petrov was the duty officer monitoring the Soviet early warning system when it reported multiple incoming US nuclear missiles. Protocol demanded he report this as a genuine attack, which would likely have triggered a retaliatory nuclear launch. Instead, he judged it was a false alarm—a decision made with incomplete information, under extreme time pressure, and against what his instruments were telling him.

Whether an AI would have made the same call depends entirely on how it was designed and what it was optimizing for:

**An AI following strict protocol** would almost certainly have reported the attack. It would have done exactly what the system specified, which was Petrov’s official duty. This is actually a significant risk with highly capable AI systems—they can be extremely good at following their instructions even when those instructions lead to catastrophic outcomes.

**An AI trained to minimize false positives** (avoiding unnecessary escalation) might have behaved like Petrov, but this would require the system to have been designed with that specific priority, which seems unlikely for a Cold War military system.

**What made Petrov’s decision remarkable** was his integration of multiple types of reasoning:

- Technical intuition (why would the US launch only five missiles?)
- Contextual awareness (geopolitical climate didn’t suggest imminent attack)
- Healthy skepticism about new, untested technology
- Willingness to trust his judgment over instruments
- Understanding that he’d be blamed either way, but chose the option that avoided nuclear war

The challenge for AI is that Petrov operated outside his formal instructions based on wisdom, context, and values that weren’t encoded in any protocol. He made himself responsible for a judgment call that required weighing catastrophic risks in both directions.

Current AI systems struggle most with exactly this kind of situation—where following instructions may lead to disaster, where context matters enormously, and where there’s no clear "right answer" derivable from training data. They’re generally not designed to second-guess their programming based on broader situational awareness.

What’s your sense—do you think this reflects something fundamental about human judgment, or is it more about how we’ve chosen to design AI systems so far?

(ii)

This is a fascinating counterfactual to consider. An AI could potentially have helped at several critical junctures, though the outcome would depend heavily on when and how it was deployed.

**Where AI might have made a difference:**

The accident stemmed from a flawed reactor design combined with operator errors during a safety test. An advanced AI monitoring system could have:

- **Prevented the test conditions**: Recognized that running the test at low power with most safety systems disabled created dangerous instability, and either refused to proceed or alerted operators more forcefully than existing alarms
- **Detected the positive void coefficient problem**: The RBMK reactor’s design flaw (where loss of cooling actually accelerated the reaction) was known but underestimated. An AI analyzing real-time neutron flux and temperature data might have caught the runaway reaction seconds earlier and initiated emergency shutdown
- **Overridden human decisions**: When operators disabled safety systems and ignored warnings, an AI with appropriate authority could have maintained those protections

**However, there were serious constraints:**

The 1986 Soviet system had limited computing power and sensor networks compared to what modern AI requires. More fundamentally, the institutional problems ran deep—the reactor design itself was flawed, and the safety culture discouraged questioning authority. An AI would have needed both the technical capability and the institutional authority to override human operators, which seems unlikely in that context.

The most realistic scenario where AI helps significantly would be one where it was deeply integrated into the reactor’s control systems with veto power over dangerous operations—essentially preventing the test from proceeding as planned.

What aspect interests you most—the technical possibilities or the institutional challenges?
